# Supplementary figures and images for: Conditional knockout of RAD51-related genes in Leishmania major reveals a critical role for homologous recombination during genome replication
Source: PLoS Genet. 2020 Jul 1;16(7):e1008828. doi: 10.1371/journal.pgen.1008828 (PMC7360064; doi:10.1371/journal.pgen.1008828)

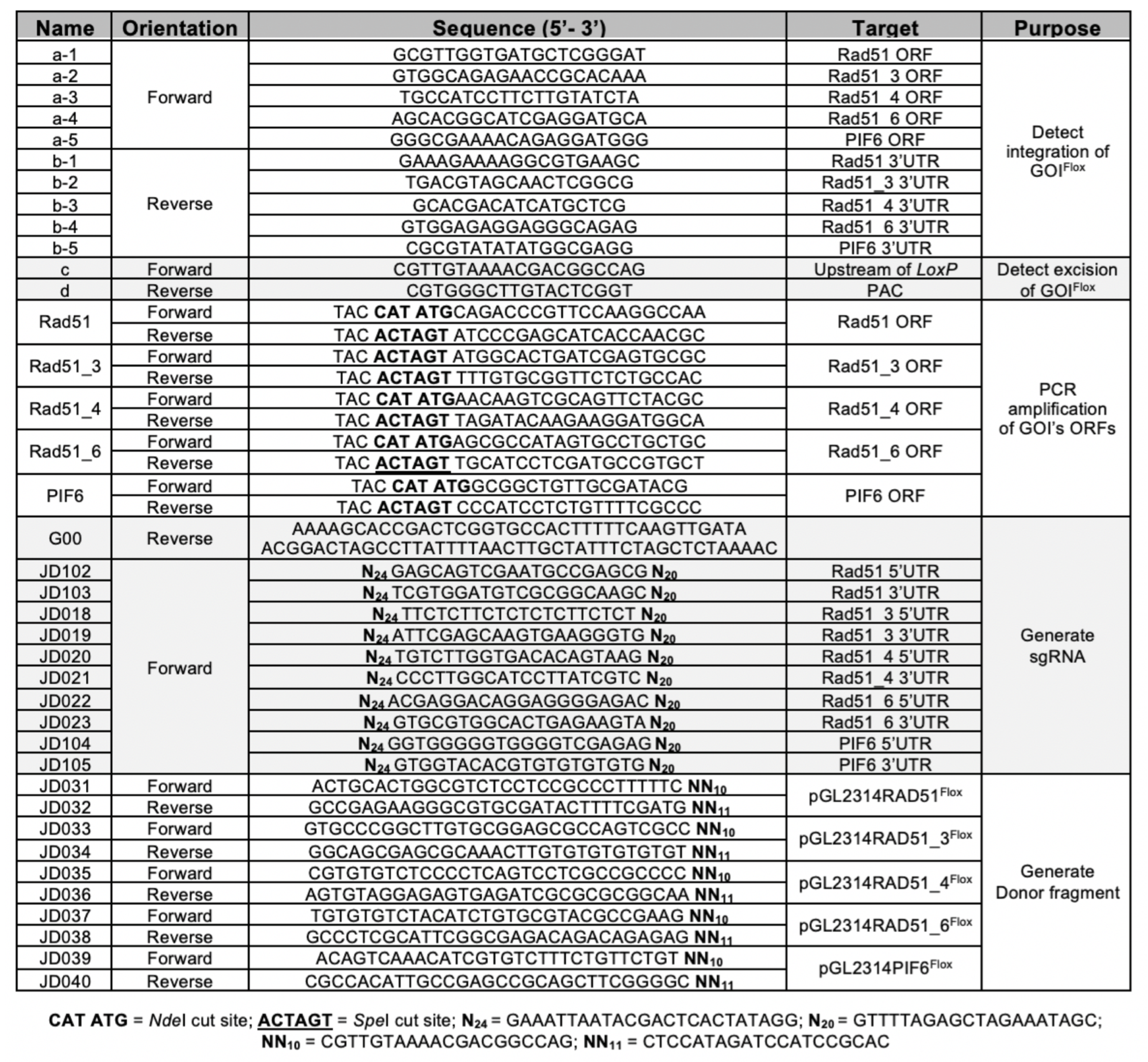

Supplement: S1 Table — (TIFF) [file pgen.1008828.s001.tiff]

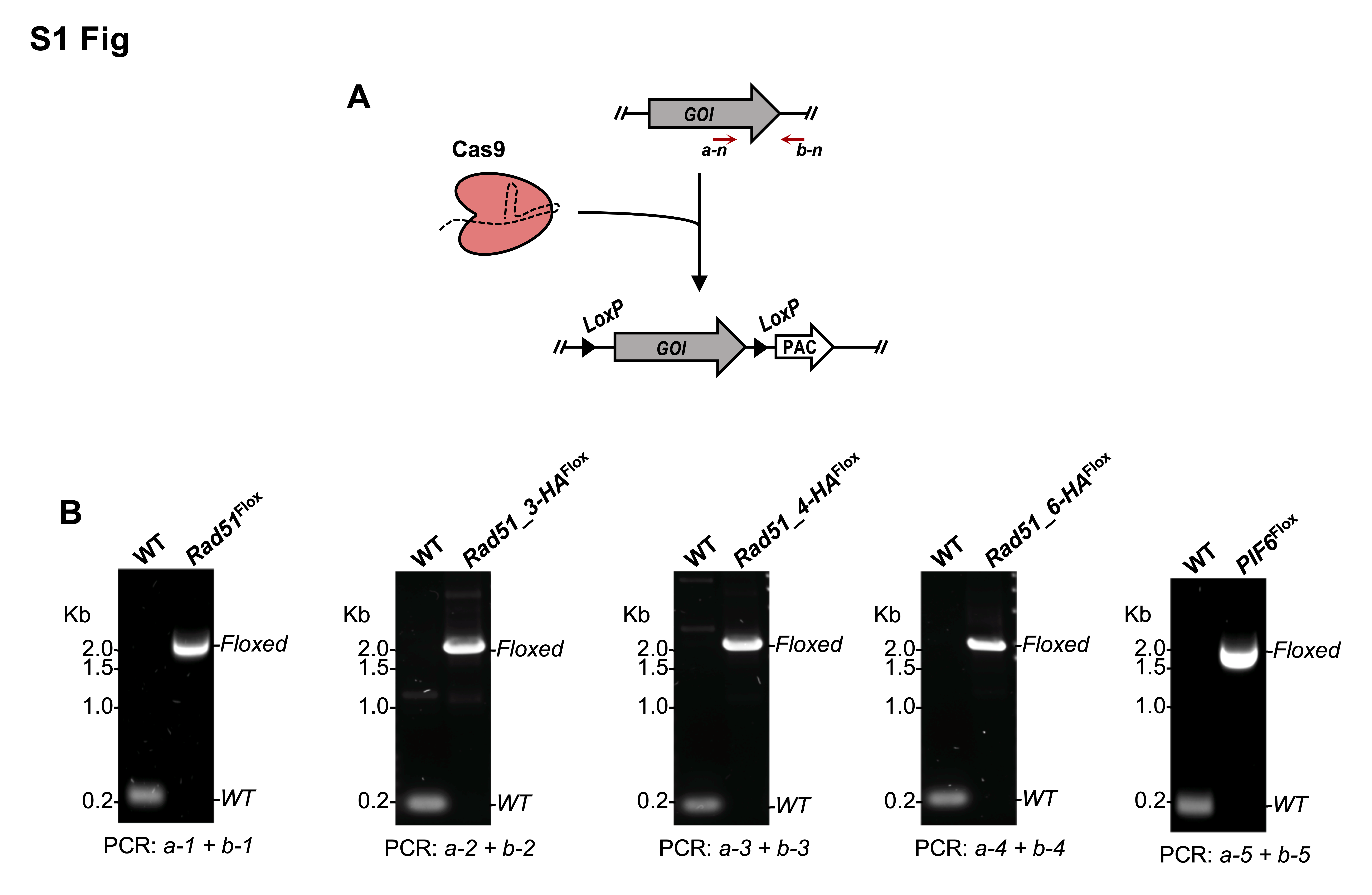

Supplement: S1 Fig — (A) Cas9 was used to replace all copies of a gene of interest (GOI) by a version of the same GOI flanked by LoxP sites (GOIFlox); (B) PCR analysis of genomic DNA extracted from the indicated cell lines; approximated annealing positions for primers a-n and b-n (where n varies from 1 to 5, indicating a distinct sequence for the targeted GOI in each cell line) are shown in (A). (TIFF) [file pgen.1008828.s002.tiff]

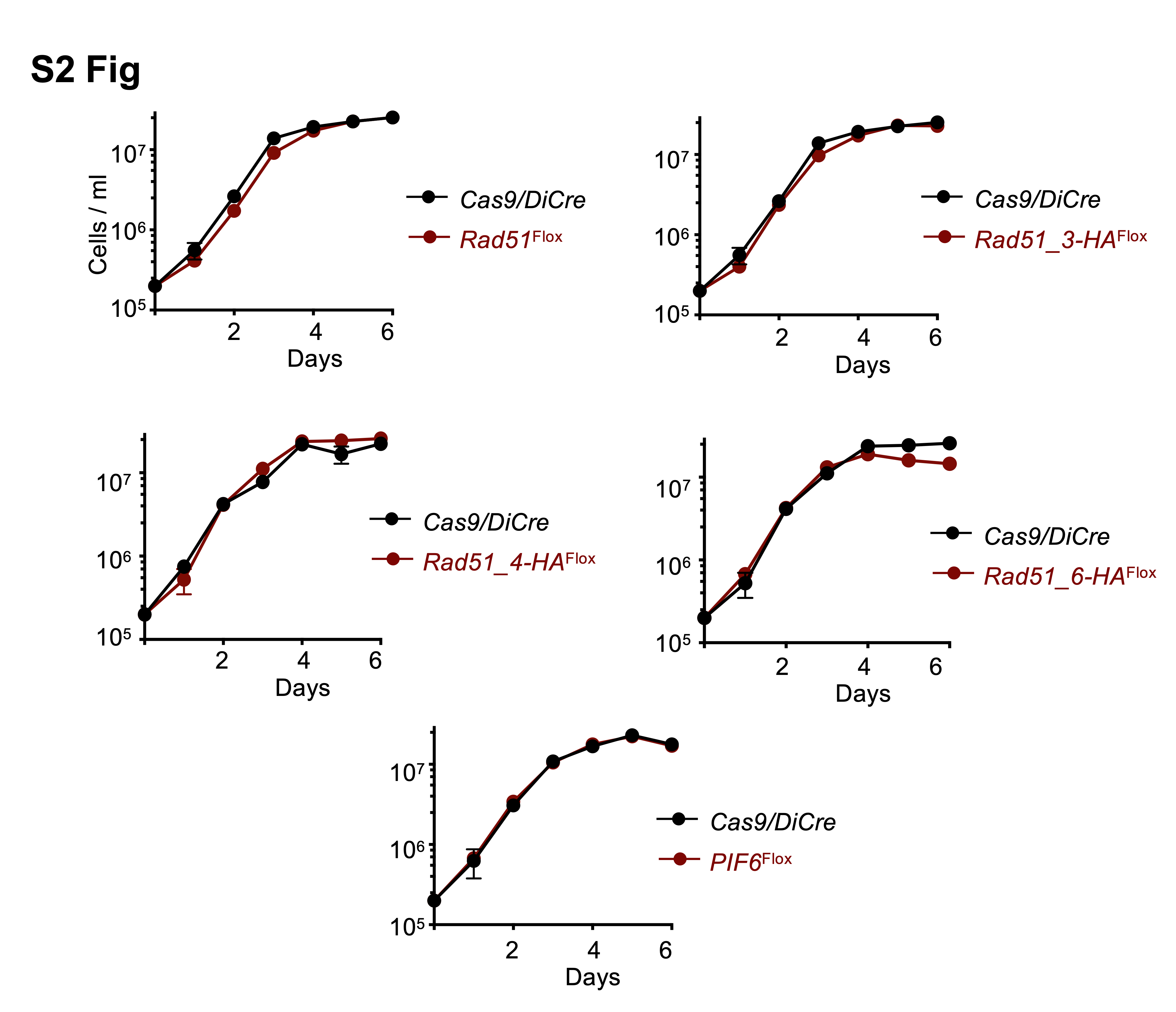

Supplement: S2 Fig — Representative growth curves of the indicated cell lines (red lines) compared to the parental cell line expressing Cas9 and DiCre (black line); growth curves were started with 2 x1 05 cells/ml; cell density was assessed every 24 h (1 day) and error bars depict standard error of the mean (S.E.M.). (TIFF) [file pgen.1008828.s003.tiff]

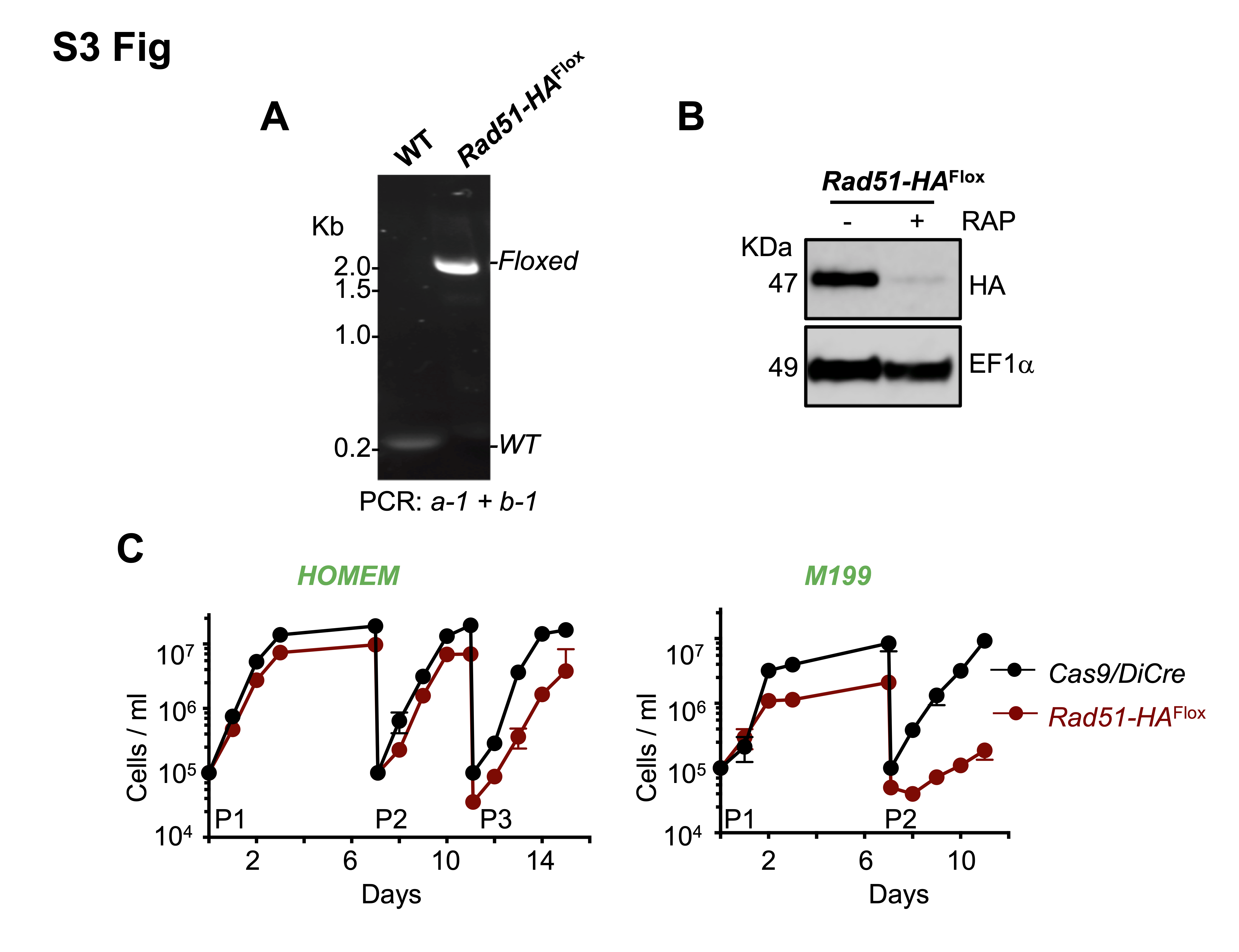

Supplement: S3 Fig — Cas9 was used to replace all copies of RAD51 by RAD51-HAFlox. (A) PCR analysis of genomic DNA from the RAD51-HAFlox cell line; approximate annealing positions for primers a-1 and b-1 are as shown in S1A Fig. (B) Western blotting analysis of whole cell extracts from RAD51-HAFlox cell line after 48 h growth without addition (-RAP) or after addition (+RAP) of rapamycin; extracts were probed with anti-HA antiserum and anti-EF1α was used as loading control. (C) Representative growth curves of RAD51-HAFlox cells (red lines) compared to the parental cell line expressing Cas9 and DiCre (black line) in both HOMEM and M199 medium; growth curves were started with 1 x 10^5^ cells/ml; cell density was assessed at the indicated days and error bars depict standard error of the mean (S.E.M.). (TIFF) [file pgen.1008828.s004.tiff]

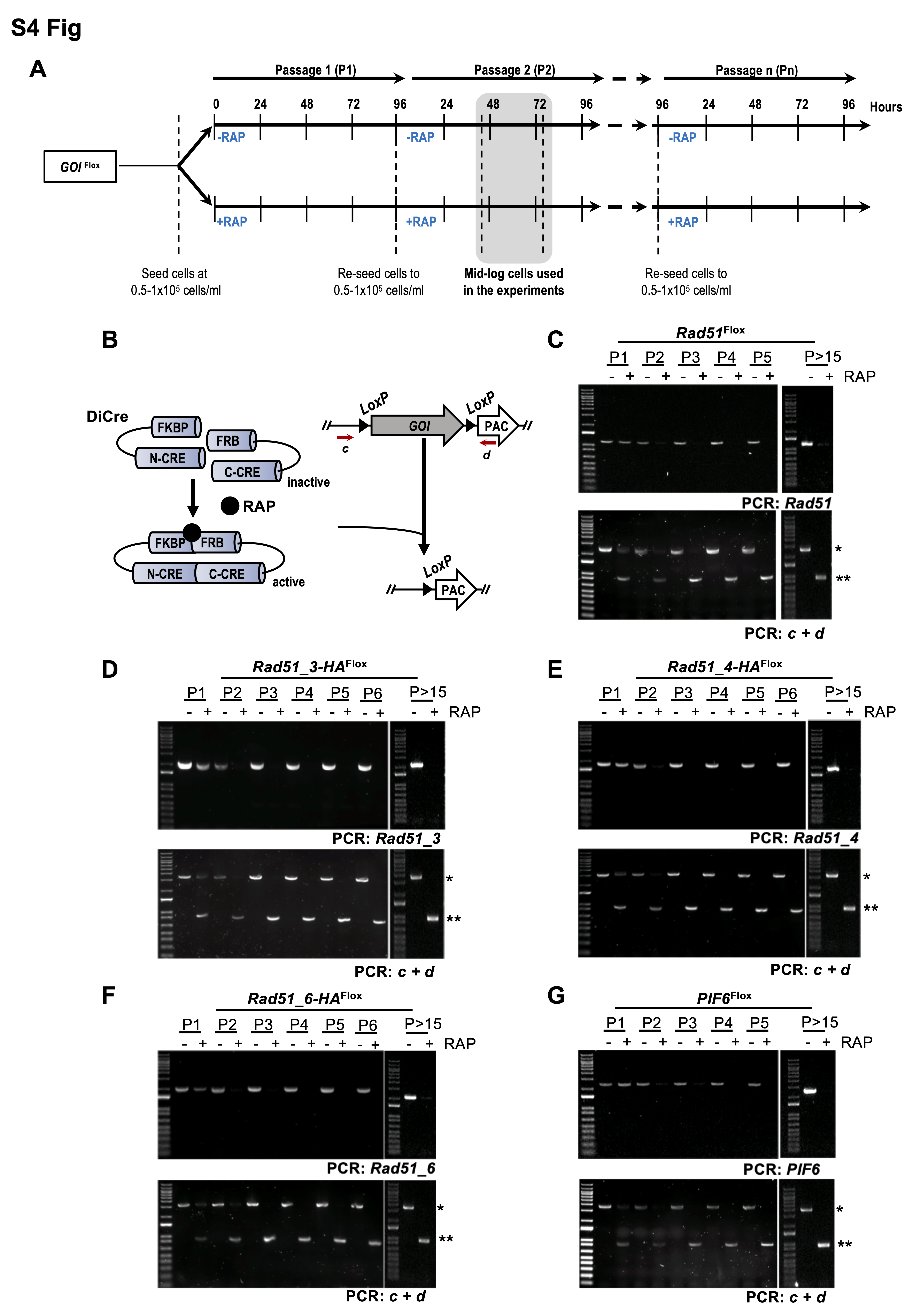

Supplement: S4 Fig — (A) Illustration of KO induction scheme; cells were seeded in medium with (+RAP) or without (-RAP) rapamycin; after 4 days (~96 h) of cultivation, cells were re-seeded, cultivated further and then diluted again; all the experiments reported here were performed in cells subjected to this induction protocol; times points indicated in the main figures refer to the second passage (P2, highlighted).(B) Illustration of GOIFlox excision catalyzed by DiCre, as induced by rapamycin. (C)-(G) PCR analysis of genomic DNA from the indicated cell lines throughout the indicated passages; DNA was extracted from cells ~72 h of each passage; approximate annealing positions for primers c and d are shown in (A); (*) and (**): GOIFlox and GOIFlox after excision, respectively. (TIFF) [file pgen.1008828.s005.tiff]

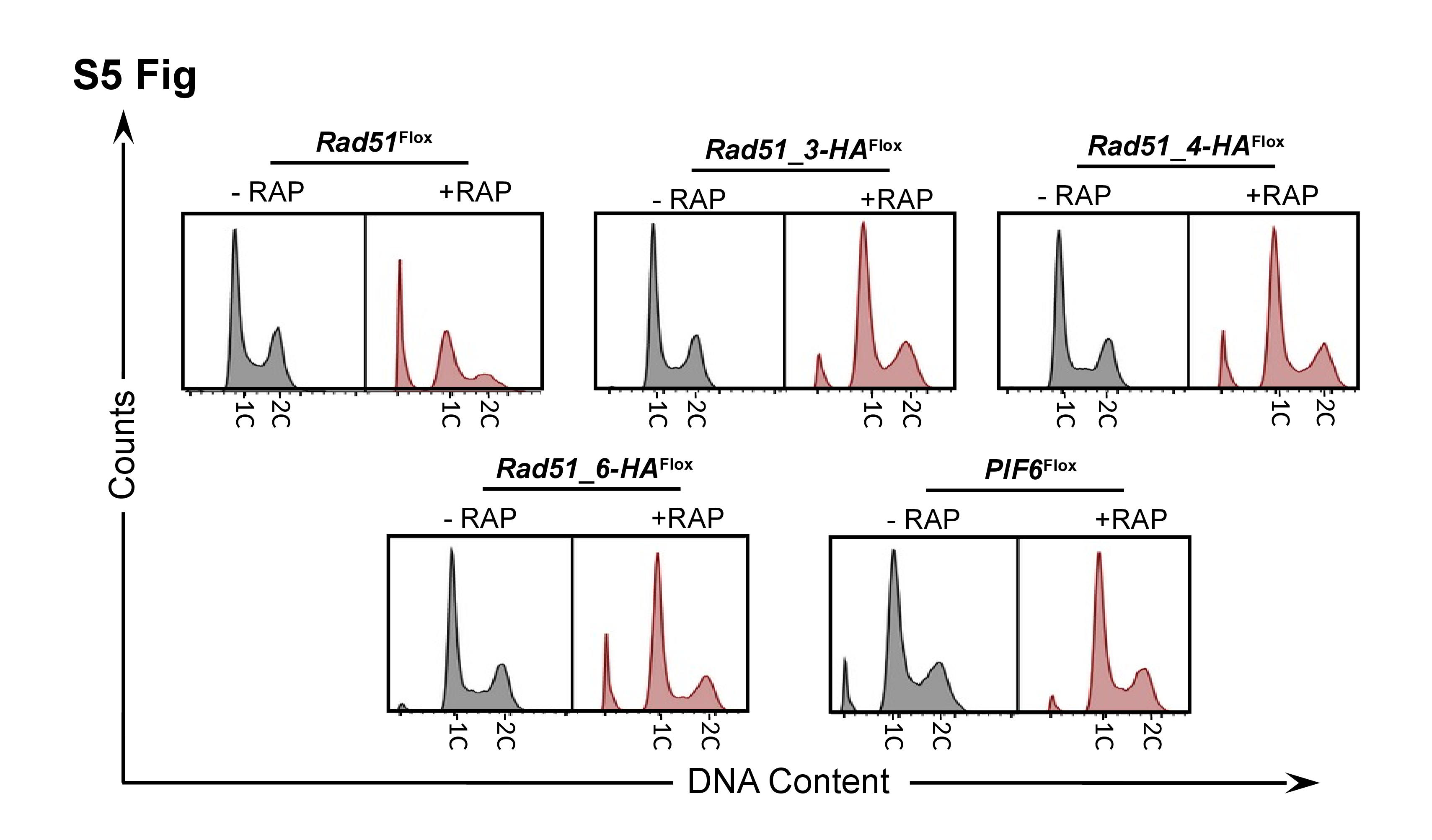

Supplement: S5 Fig — Representative histograms from FACS analysis to determine the distribution of cell populations according to DNA content in cells kept in culture for more than 15 passages; 30,000 cells were analysed per sample; 1C and 2C indicate single DNA content (G1) and double DNA content (G2/M), respectively. (TIFF) [file pgen.1008828.s006.tiff]

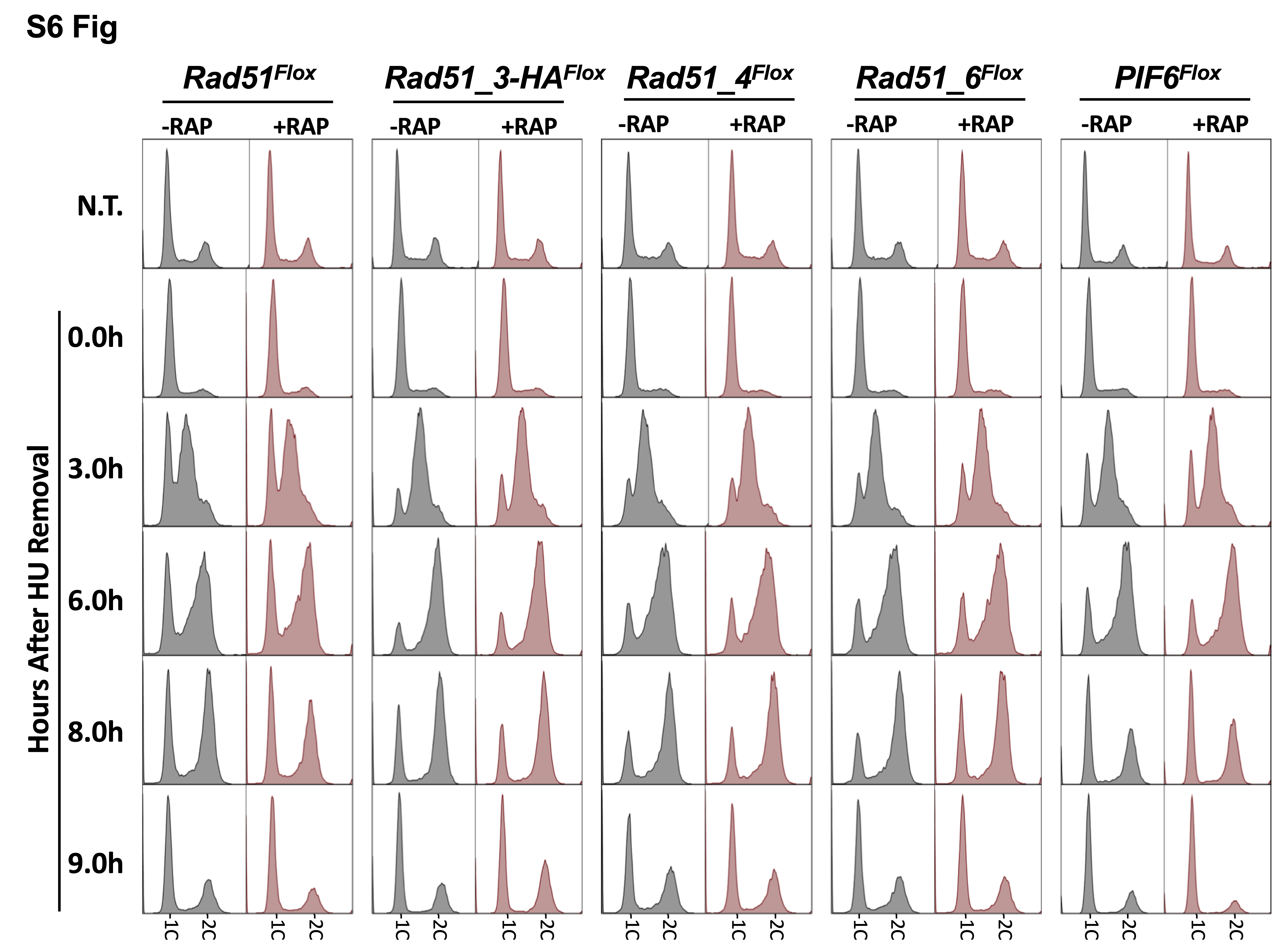

Supplement: S6 Fig — The indicated cell lines were left untreated (N.T.) or treated for 8 h with 5 mM HU and then re-seeded in HU-free medium; cells were collected at the indicated time points after HU removal, fixed, stained with Propidium Iodide, and analysed by FACS; 1C and 2C indicate single DNA content (G1) and double DNA content (G2/M), respectively. (TIFF) [file pgen.1008828.s007.tiff]

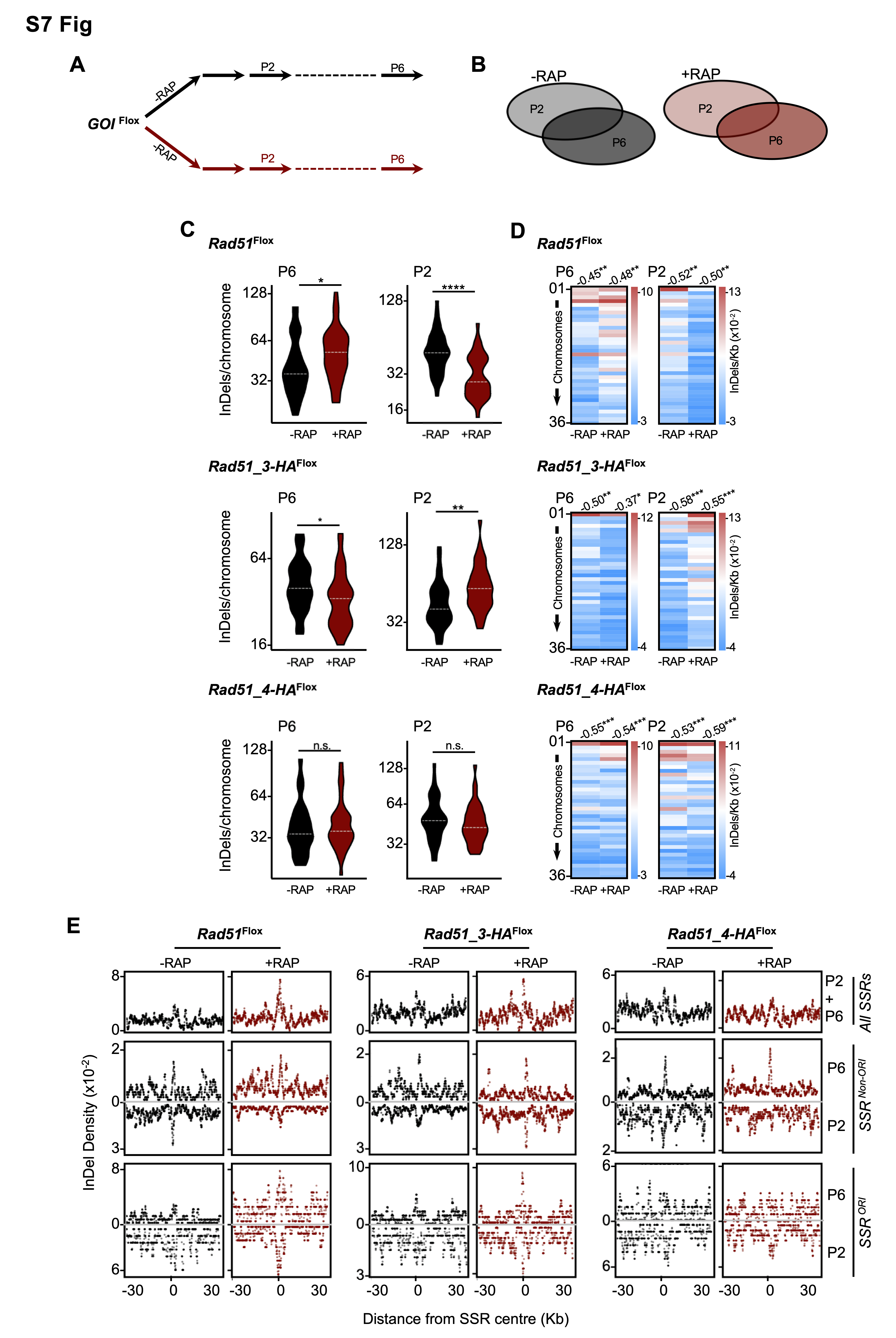

Supplement: S7 Fig — (A) GOIFlox cell lines were grown in the absence (-) or presence (+) of Rapamycin (RAP). Genomic DNA was extracted at P2 and P6 and subjected to deep sequencing. (B) InDels relative to the reference genome were identified. Events common to P2 and P6, with or without RAP, were discarded. Events exclusively found in P2 or P6 were considered for the following analysis. (C) Quantification of the number of new InDels detected inP2 and P6; data are represented as violin plots, where shape indicates the distribution of pooled data and horizontal dotted white lines indicate the median; differences were tested with Mann-Whitney test; * P<0.05, **P<0.005 and ***P<0.001 (D) Heatmaps representing density of new InDels (InDels/Kb) detected in the indicated passages; numbers at the top of each row indicate Pearson correlation between InDel density and chromosome size; when correlation is significant, it is indicated by * P<0.05, **P<0.005 and ***P<0.001. (E) Metaplots of normalized density of InDels (InDels/Kb) in passages P2 and P6is plotted +/- 30 Kb around the centre of either SSRORI (n = 36) or SSRnon-ORI (n = 95) for the indicated cell lines. (TIFF) [file pgen.1008828.s008.tiff]

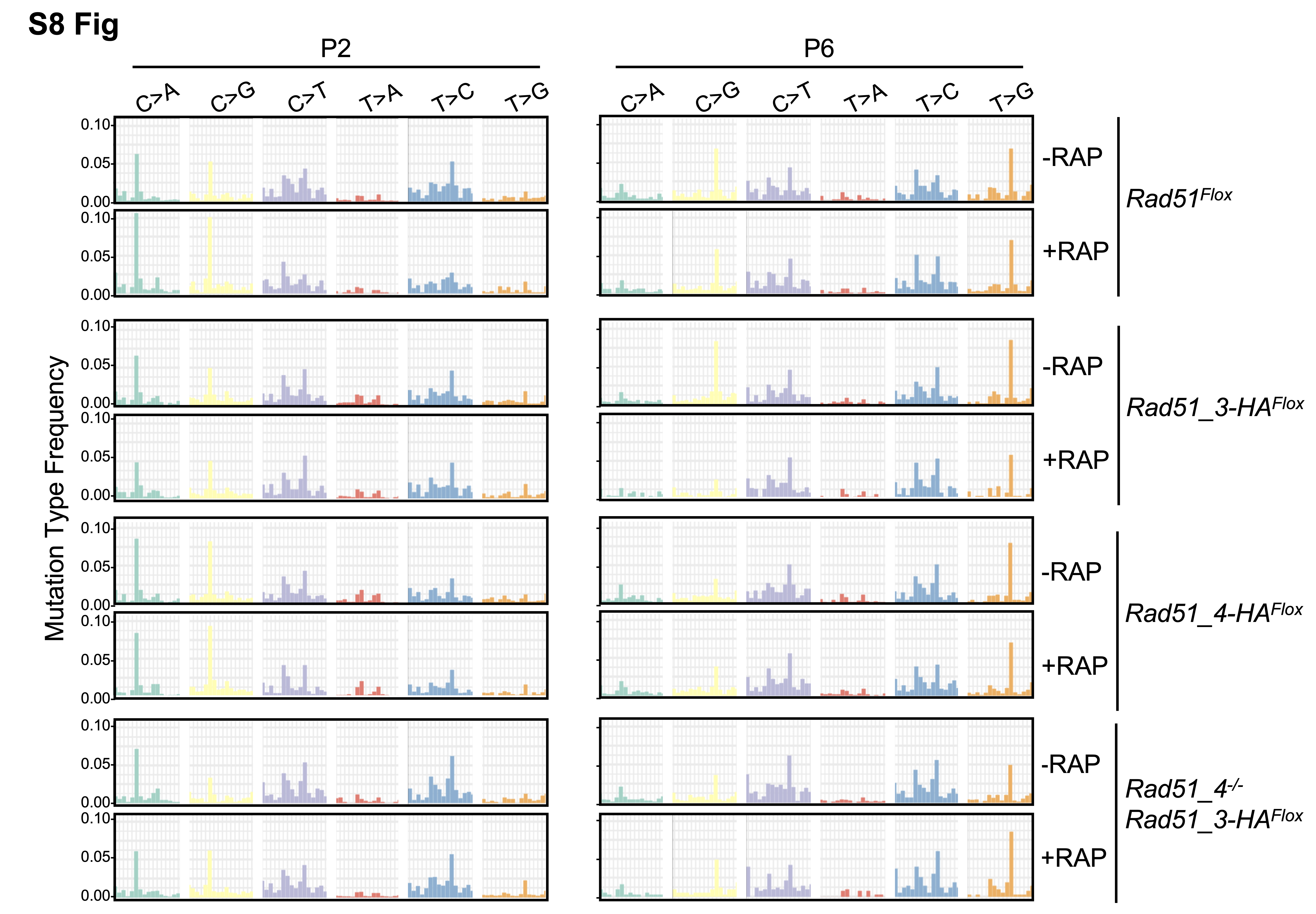

Supplement: S8 Fig — SNPs were ordered by class (C>A/G>T, C>G/G>C, C>T/G>A, T>A/A>T, T>C/A>G, T>G/A >C) and subsequently subclassified according to immediate flanking sequence: 5′ base (A, C, G, T) before 3′ base (A, C, G, T). (TIFF) [file pgen.1008828.s009.tiff]

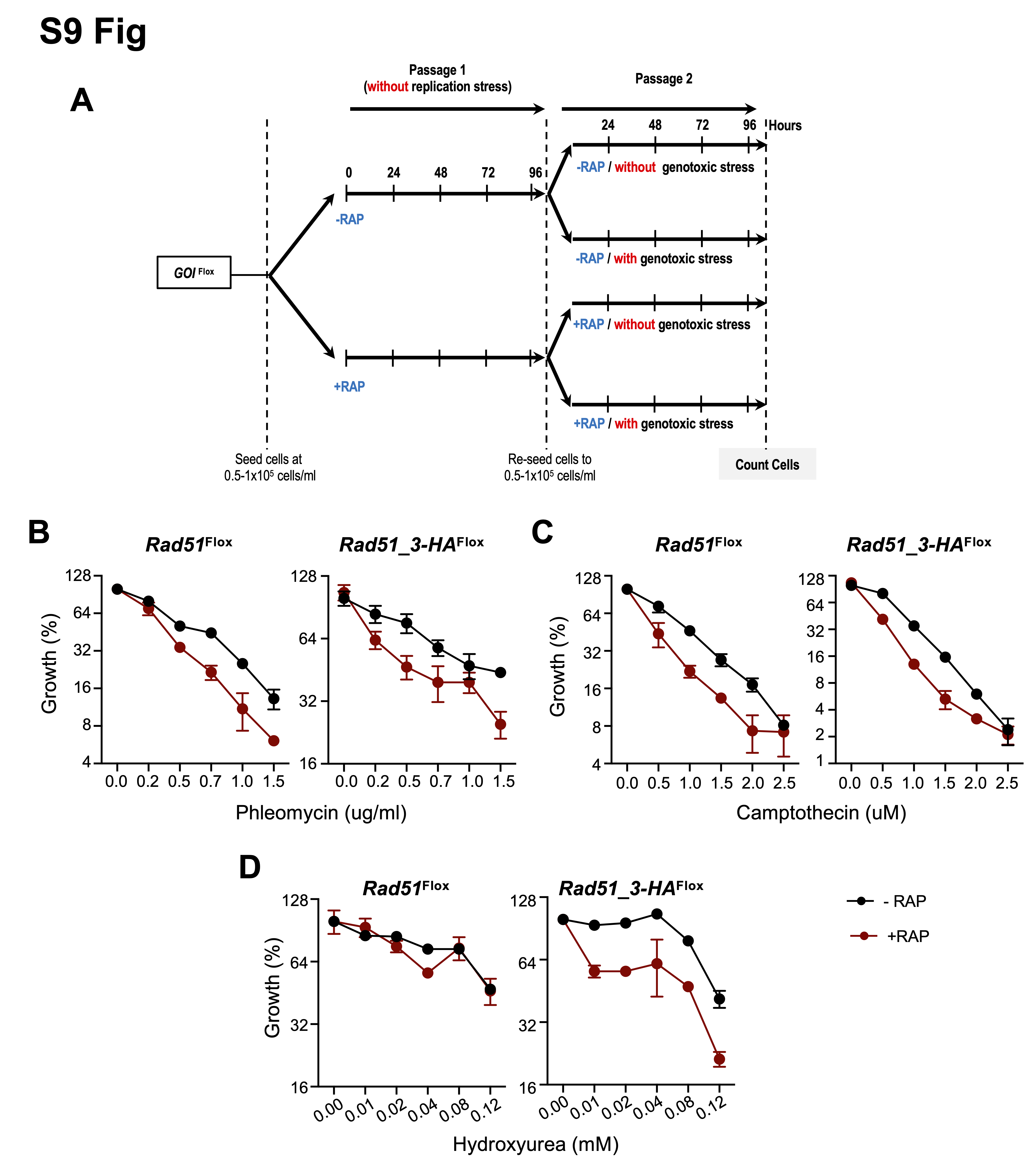

Supplement: S9 Fig — (A) Experimental design to evaluate resistance to genotoxic agents as shown in (B-D); cells were seeded in medium with (+RAP) or without (-RAP) rapamycin, in the absence of any genotoxic drug; after 96 h of growth, cells were re-seeded in medium with or without genotoxic agents at various concentrations; after 96 h growth (P2), cell density in each condition was determined. (B–D) Relative growth of cells incubated with the indicated concentration of the indicated genotoxic agents, during P2; growth in each concentration is expressed as a percentage of proliferation relative to cells cultivated without the genotoxic drugs; error bars depict SD. (TIFF) [file pgen.1008828.s010.tiff]

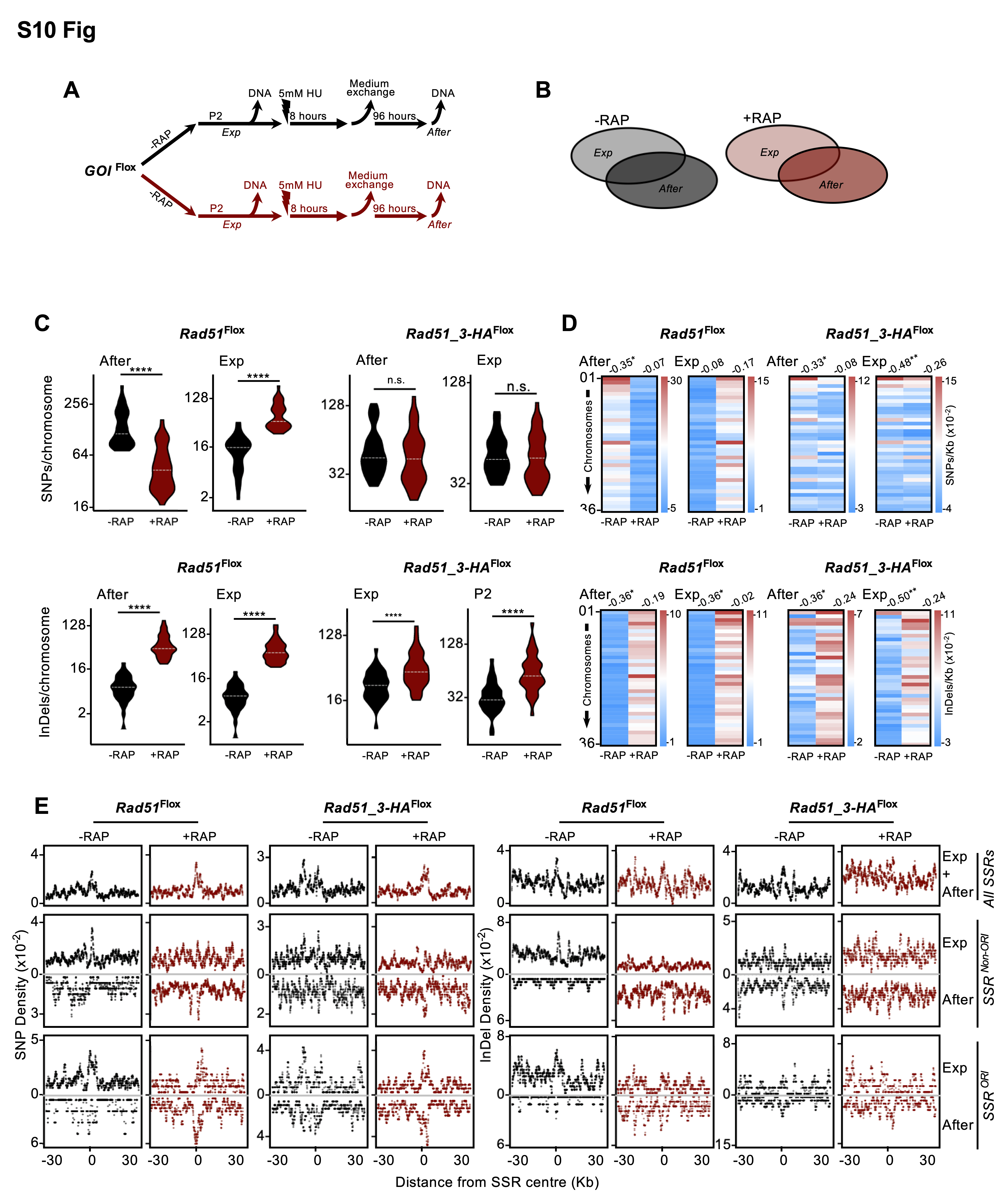

Supplement: S10 Fig — (A) GOIFlox cell lines were grown in the absence (-) or presence (+) of Rapamycin (RAP) for 2 passages (P2). Genomic DNA was extracted from exponentially (Exp) growing cells in P2. Then, cells were incubated with 5 mM HU for 8 h. Cells were re-seeded in HU-free medium and after (After) 96 h genomic DNA extracted and subjected to deep sequencing. (B) SNPs and InDels relative to the reference genome were identified. Events common to Exp and After cells were discarded. Only events exclusively found in Exp or After were considered for the following analysis. (C) Quantification of the number of new InDels detected in passages Exp and After. Data are represented as violin plots, where shape indicates the distribution of pooled data and horizontal dotted white lines indicate the median; differences were tested with Mann-Whitney test; * P<0.05, **P<0.005 and ***P<0.001. (D) Heatmaps representing density of SNPs (SNPs/Kb) and InDels (InDels/Kb) detected in Exp and After; numbers at the top of each row indicate Pearson correlation between SNPs density or InDels density and chromosome size; when correlation is significant, it is indicated by * P<0.05, **P<0.005 and ***P<0.001. (E) Metaplots of normalized density of new SNPs (SNPs/Kb) and new InDels (InDels/Kb), respectively, detected 96 h after cells were released from HU treatment are plotted +/- 30 Kb around the centre of either SSRORI (n = 36) or SSRnon-ORI (n = 95) for the indicated cell lines. (TIFF) [file pgen.1008828.s011.tiff]

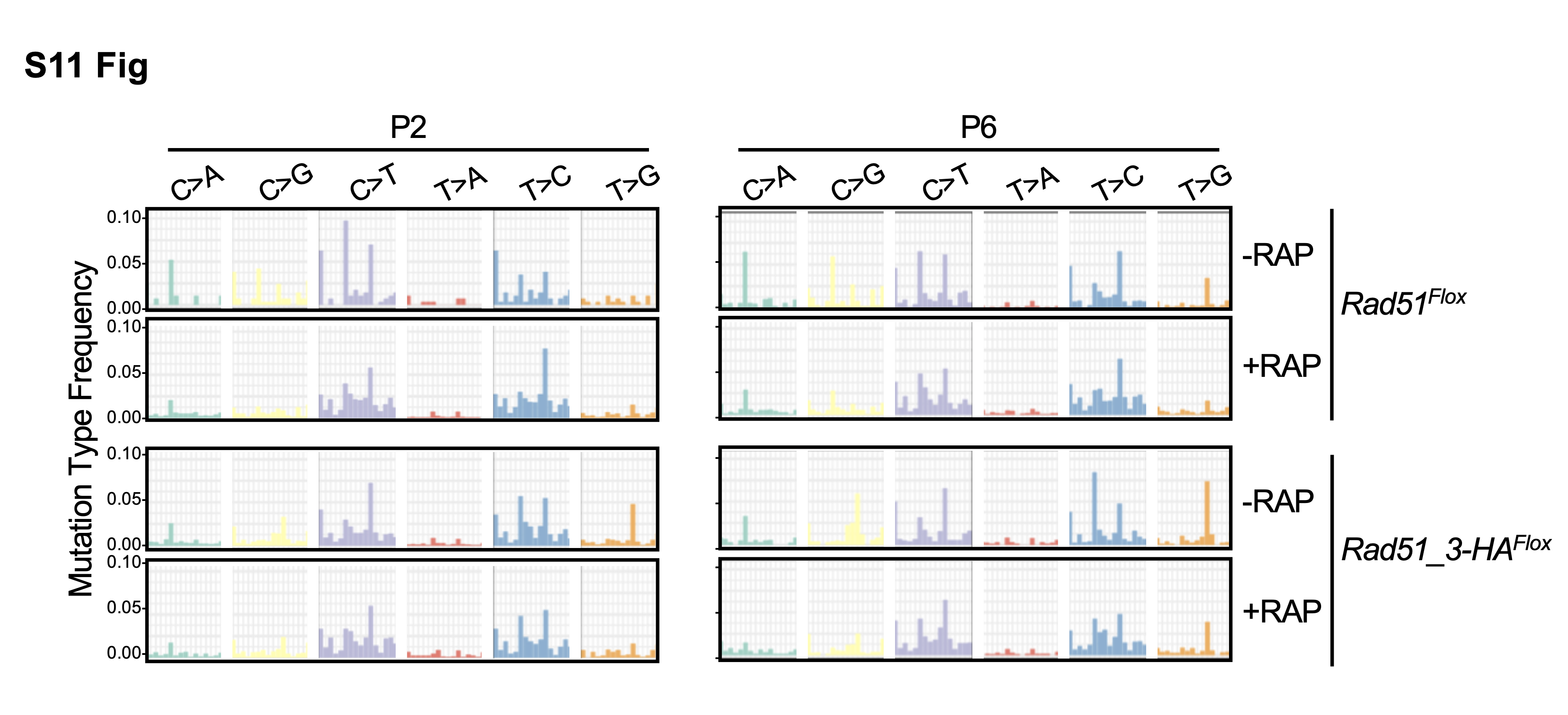

Supplement: S11 Fig — SNPs were ordered by class (C>A/G>T, C>G/G>C, C>T/G>A, T>A/A>T, T>C/A>G, T>G/A >C) and subsequently subclassified according to immediate flanking sequence: 5′ base (A, C, G, T) before 3′ base (A, C, G, T). (TIFF) [file pgen.1008828.s012.tiff]

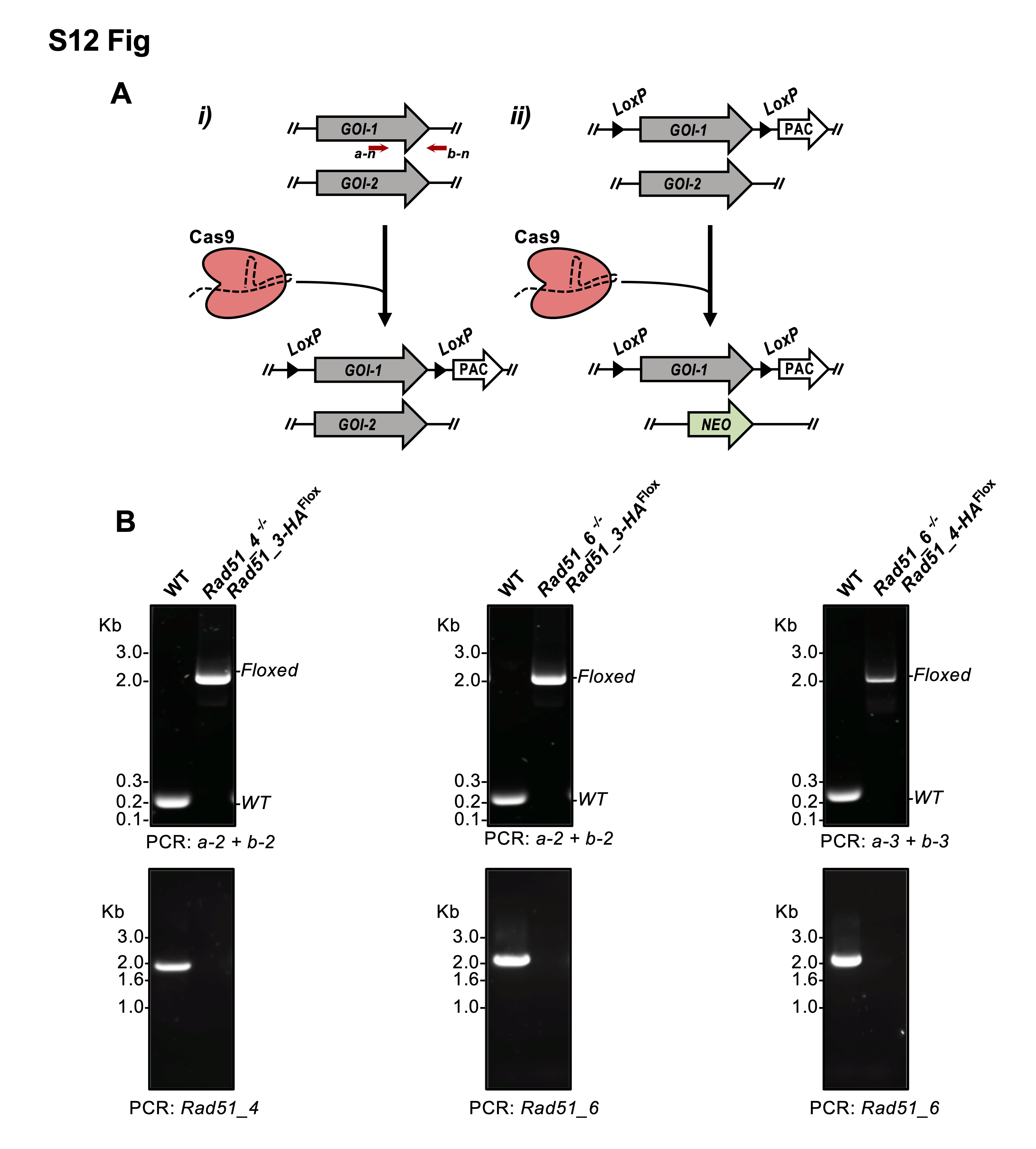

Supplement: S12 Fig — (A) i) Cas9 was used to replace all copies of a gene of interest (GOI-1) by a version of the same GOI flanked by LoxP sites (GOI-1Flox); ii) in the same cell line, Cas9 was used to replace all copies of another gene of interest (GOI-2) by a Neomycin resistance gene (NEO); (B) PCR analysis of genomic DNA extracted from the indicated cell lines; approximate annealing positions for primers a-n and b-n (where n varies from 1 to 5, indicating a distinct sequence for the targeted GOI in each cell line) are shown in (A). (TIFF) [file pgen.1008828.s013.tiff]

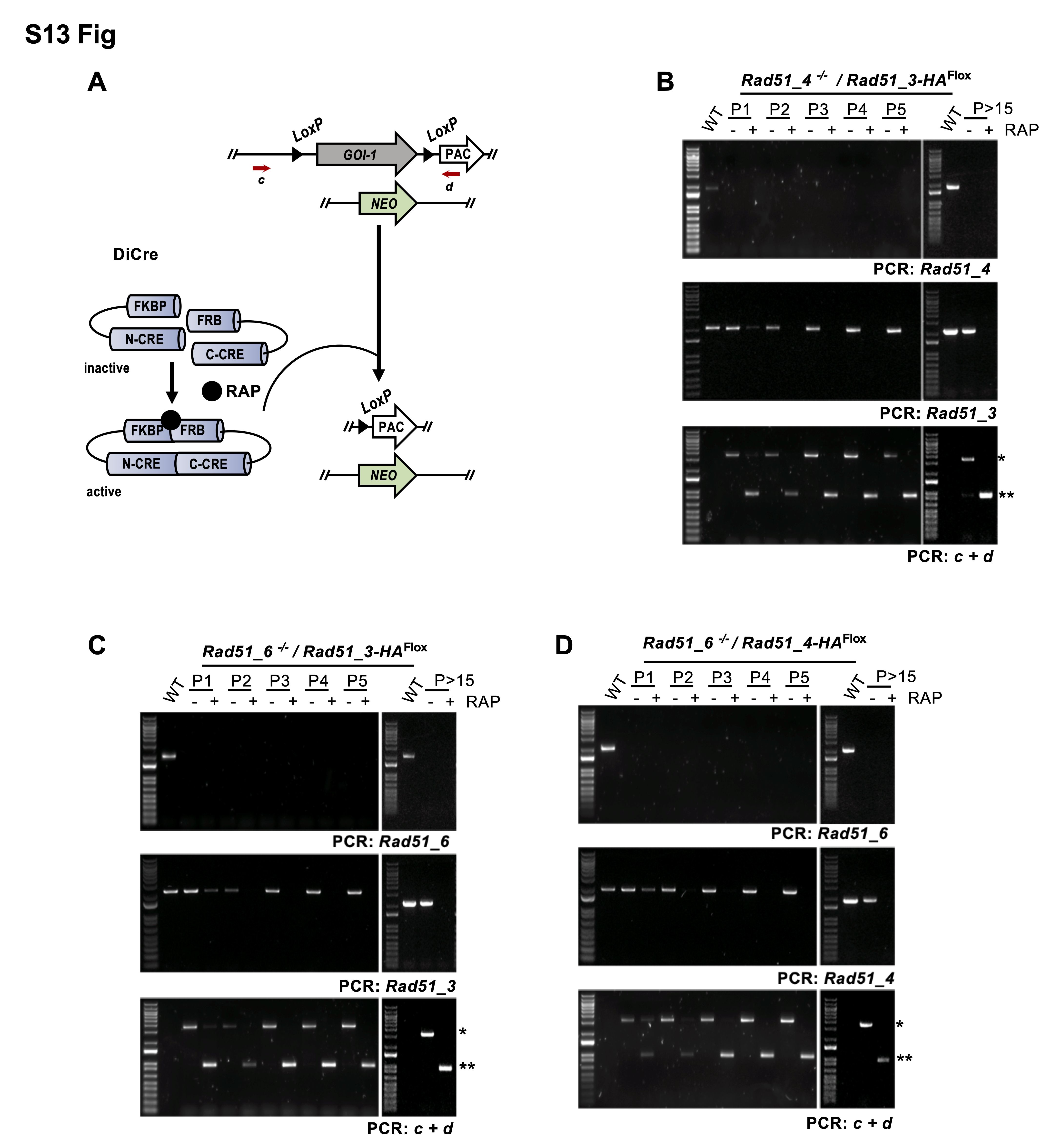

Supplement: S13 Fig — Induction was performed as depicted in S3A Fig. (A) Illustration of GOIFlox excision catalyzed by DiCre, as induced by rapamycin, to generate double KO cells. (B)—(D) PCR analysis of genomic DNA from the indicated cell lines throughout the indicated passages; approximate annealing positions for primers c and d are shown in (A); (*) and (**), GOIFlox and GOIFlox after excision, respectively. (TIFF) [file pgen.1008828.s014.tiff]

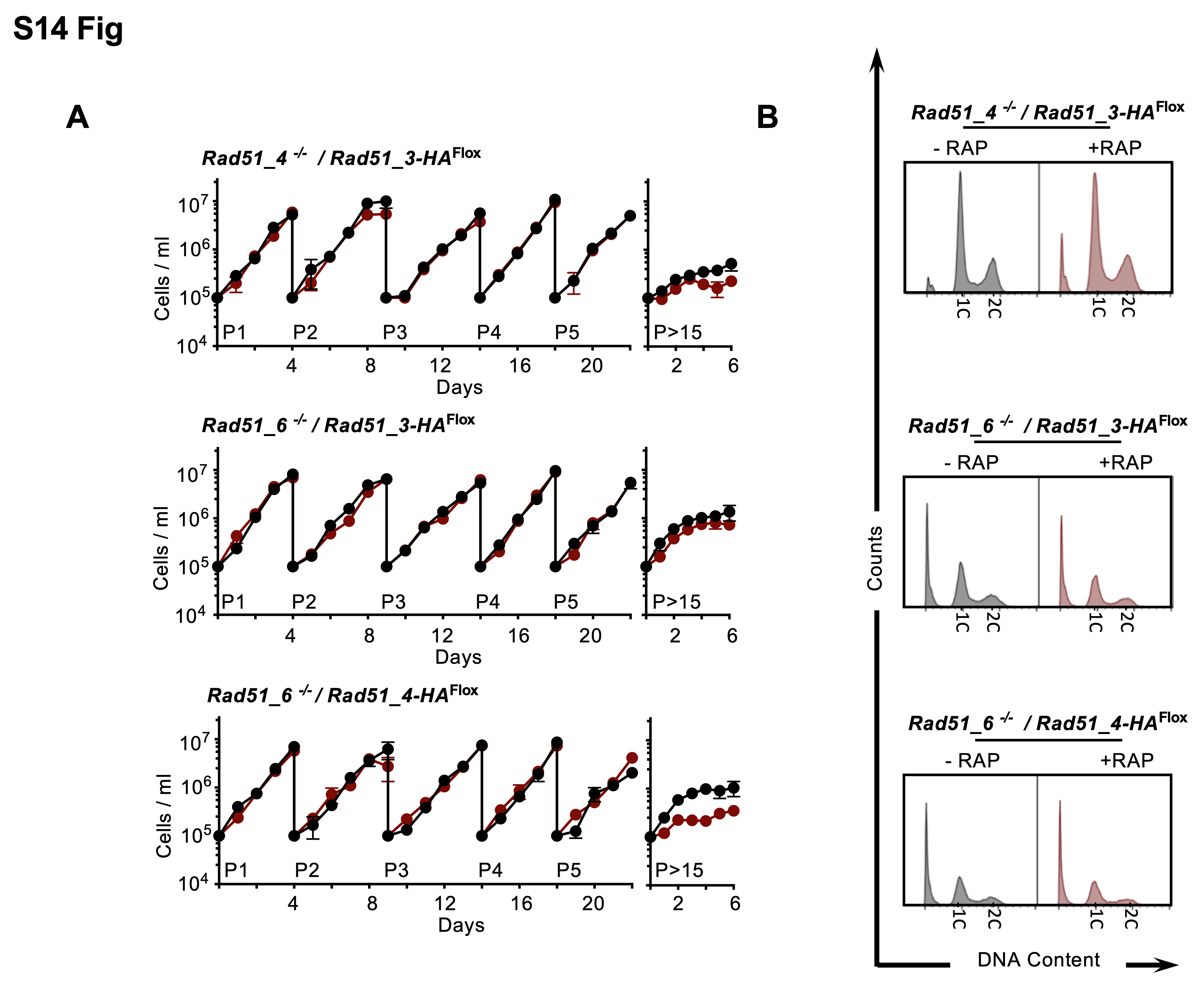

Supplement: S14 Fig — (A) Representative growth curves of the indicated cell lines in the presence or absence of RAP; cells were seeded at 105 cells/ml in day 0 and re-seeded every 4–5 days to complete five passages (P1 to P5); growth profile was also evaluated after cells were kept in culture for more than 15 P (>P15); cell density was assessed every 24 h and error bars depict standard error of the mean (S.E.M.). (B) Representative histograms from FACS analysis to determine the distribution of cell population according to DNA content in cells kept in culture for more than 15 P; 30,000 cells were analysed per sample; 1C and 2C indicate one DNA content (G1) and double DNA content (G2/M), respectively. (TIFF) [file pgen.1008828.s015.tiff]

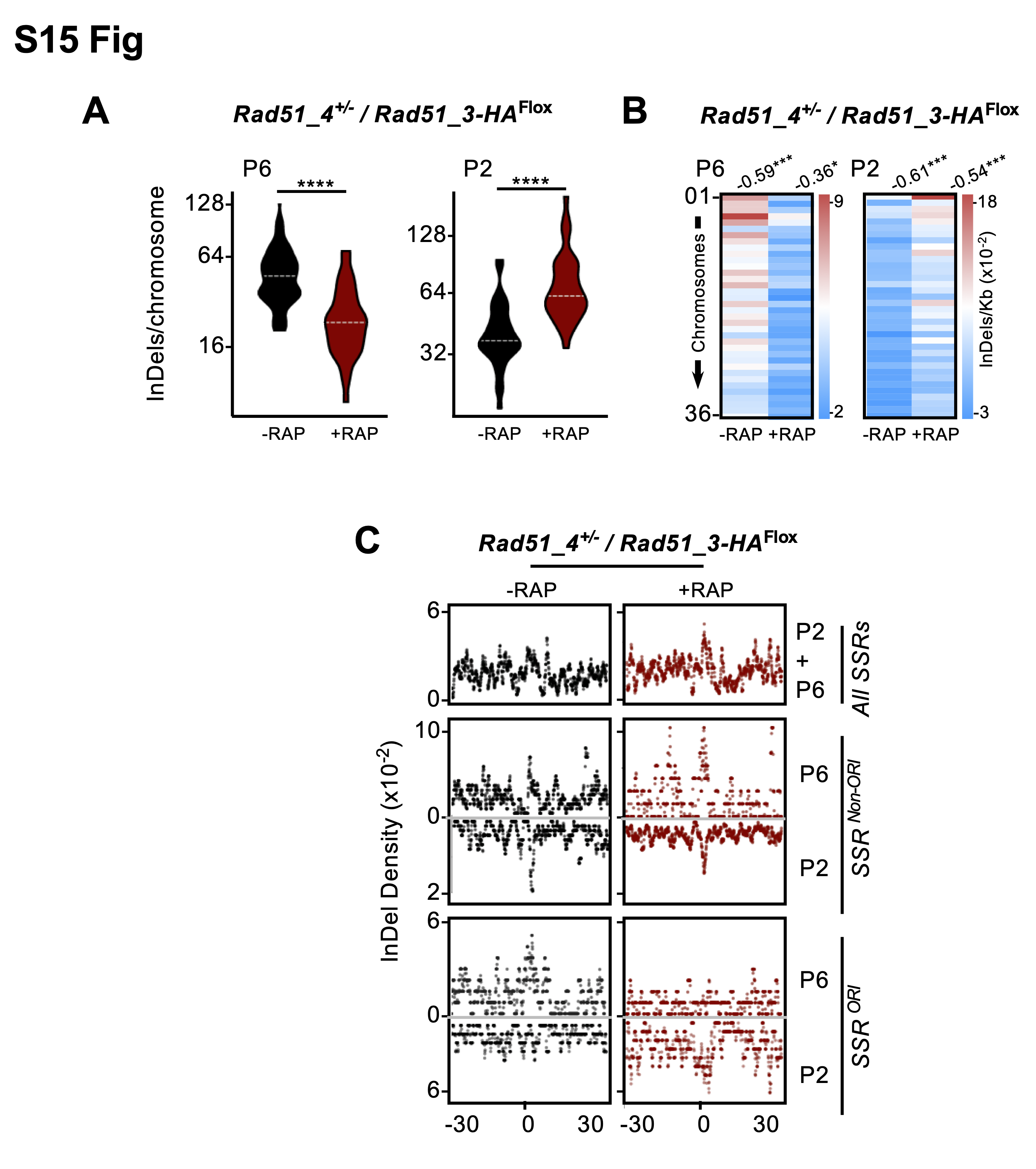

Supplement: S15 Fig — (A) Quantification of the number of new InDels detected after P6 and P2; data are represented as violin plots, where shape indicates the distribution of pooled data and horizontal doted white lines indicate the median. (B) Heatmaps representing density of new InDels (InDels/Kb) detected after 4 P in each chromosome. (C) Metaplots of normalized density of new InDels (InDels/Kb) after 4P are plotted +/- 30 Kb around the centre of either SSRORI (n = 36) or SSRnon-ORI (n = 95) for the indicated cell lines. (TIFF) [file pgen.1008828.s016.tiff]

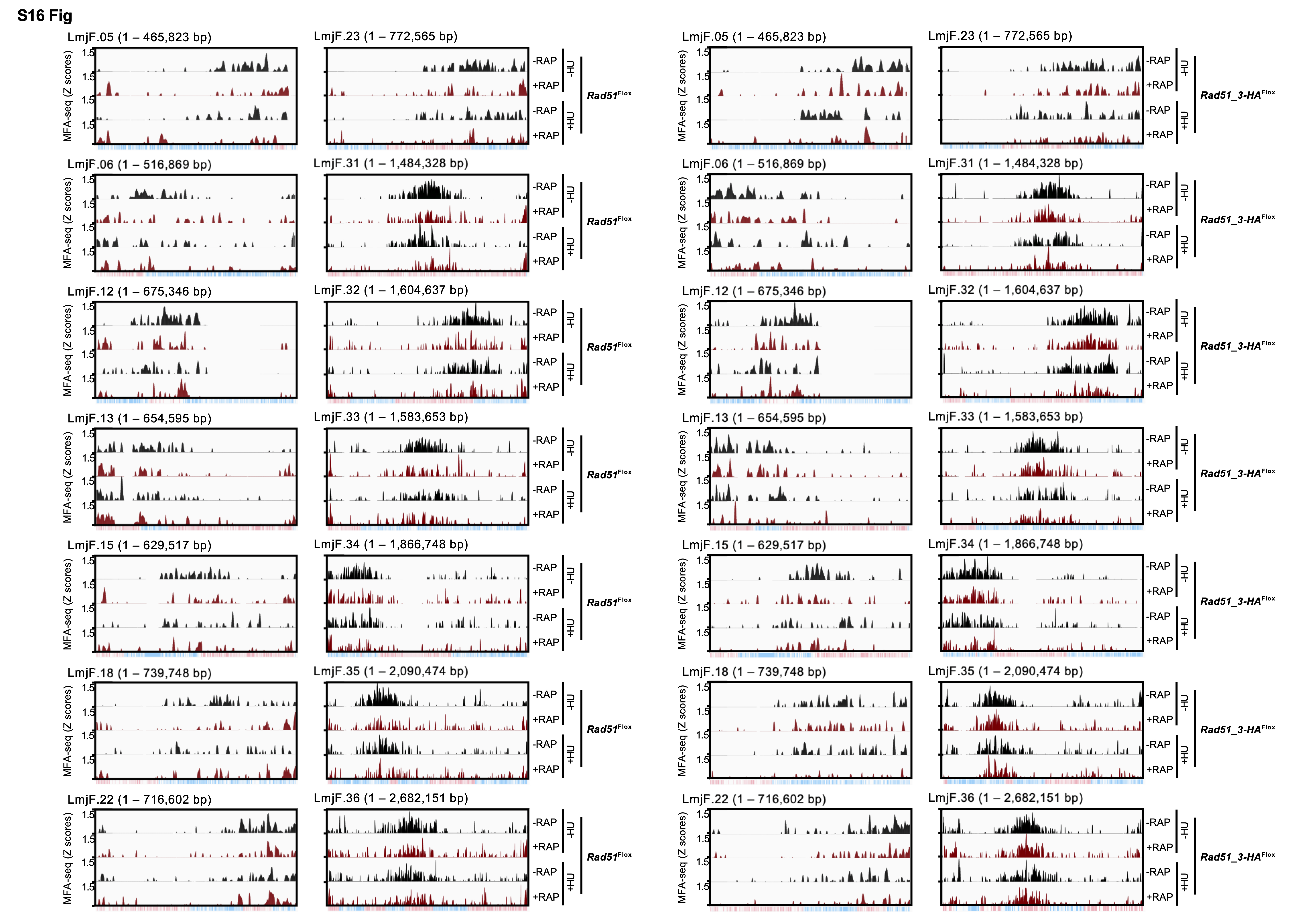

Supplement: S16 Fig — Graphs show the distribution of sites of DNA synthesis initiation across the indicated chromosomes in the indicated cell lines, in each case grown in the absence (-RAP) or the presence (+RAP) of rapamycin. MFA-seq profiles are also shown for cells after incubation with 5 mM HU for 8 h. MFA-seq is represented by Z-scores across the chromosomes, calculated by comparing read depth coverage of DNA from exponentially growing cells relative to stationary cells; the bottom track for each chromosome displays coding sequences, with genes transcribed from right to left in red, and from left to right in blue. (TIFF) [file pgen.1008828.s017.tiff]
