# Supplementary material for: Conditional knockout of RAD51-related genes in Leishmania major reveals a critical role for homologous recombination during genome replication
Source: PLoS Genet. 2020 Jul 1;16(7):e1008828. doi: 10.1371/journal.pgen.1008828 (PMC7360064; doi:10.1371/journal.pgen.1008828)
Supplement: Attachment — Submitted filename: Response to reviewers.pdf [file pgen.1008828.s019.pdf]

Dear Marc,

Thank you and the three reviewers for considering our submission PGENETICS-D-19-01903, *Conditional knockout of RAD51-related genes in Leishmania major reveals a critical role for homologous recombination during genome replication*. The thoughtful comments of the reviewers were very helpful and, in responding, we believe the paper has been improved. Below is a summary of the new data we have added, as well as responses to each of the reviewers' comments. We hope that this new submission is acceptable for publication.

Best wishes,

Richard

New data:

Fig.1B. To avoid confusion between HA-tagged RAD51, which we find to have impaired function (see below, Fig.S3), and untagged RAD51, we have added RT-PCR to show loss of the latter after DiCre induction.

Fig.1F. We have added growth curves of cells after DiCre excision in M199 medium, where we see earlier arising and potentially stronger growth defects, showing that loss of RAD51-like factors can cause pronounced problems in *Leishmania* that appear somewhat masked by growth in HOMEM medium (reviewers #1 and #2).

Figs.3C, 3D, 3E and 3F. We have improved analysis of SNP accumulation before and after DiCre induction. 3C shows the approach: we measured SNPs in both induced (RAP+) and non-induced (RAP-) cells at passage 2 and 4, comparing to the reference genome, and discarding mutations common to the two passages in RAP+ and – (data in 3D-F), which likely represent differences between the genomes of the cells used and the *L. major* reference. In this way we provide a measure of the levels of new mutations that arise only during growth with and without loss of individual HR factors. We have also assessed the significance of the data (reviewer #3).

Figs.5C, 5D, 5E and 5F. As in Fig.3, improved SNP analysis, but here examining the double RAD51-3 and RAD51-4 mutant.

Fig.6. Chromosome-specific and global analysis of MFA-seq before and after DiCre-induced loss of RAD51 or RAD51-3, but now including the effect of HU treatment (new panels in A, B and C; reviewers #2 and #3)

Fig.S3. To avoid confusion, we have moved analysis of RAD51-HA^{flox} cells to supplementary, since these were not used for most experiments due to addition of an epitope tag to RAD51 resulting in impaired growth relative to parental cells even prior to gene excision (reviewers #1 and #3).

Fig.S7 (previously S6). The same improved mutation analysis for individual HR mutants as in Fig.3 but analyzing InDels rather than SNPs.

Fig.S8 Mutation signature at SNPs with and without DiCre excision (reviewer #1).

Fig.S10. A similar mutation analysis for individual HR mutants as in Fig.3, but here analyzing the effect of treatment with HU, and with a more complete explanation of the approach (Reviewer #2).

Fig.S11. Analysis of mutation signature at SNPs, as in Fig.S8, but here after addition of HU (reviewers #1 and #2).

Fig.S15 (previously S12). The same improved mutation analysis for RAD51-3 and RAD51-4 double mutants as in Fig.5 but analyzing InDels rather than SNPs.

Fig.S16 (was S13). MFA-seq before and after DiCre-induced loss of RAD51 and RAD51-3 but now including the effects of HU treatment (reviewer #3).

Responses to reviews

Reviewer #1: PGENETICS-D-19-01903

In this paper, Damasceno and colleagues scrutinized the biological consequences of conditional ablation of RAD51 and three RAD51-related proteins in Leishmania major. Loss of RAD51 or RAD51-3, but not RAD51-4 or RAD51-6, impairs DNA synthesis by affecting the initiation of DNA replication at the main origins. Interestingly, ablation of RAD51, RAD51-3, and RAD51-4 genes results in genome-wide mutagenesis. The authors also report that RAD51 plays an unexpected role for proper activation of DNA replication origins. The conclusions are drawn using several complementary approaches. The tools and conditional gene knockouts described here will be useful for the Leishmania major community.

Specific comments:

1. I have difficulty to reconcile the growth curves (Fig. 1E) to the DNA replication defects observed in Fig.

To attempt to explain this discrepancy, we have added data for growth DiCre-induced cells in a different medium, M199, where earlier arising and potentially stronger growth defects are seen after loss of any RAD51-related gene, but not PIF6 (new Fig. 1F). These data suggest that HOMEM is a more 'forgiving' media for cells with impaired cellular functions, including reduced DNA synthesis and increased levels of endogenous genome damage (also reviewer #2). What features of the two media underly this phenotypic difference is unclear. We have added the following text:

'*In fact, when grown in M199 medium, growth defects were observed more rapidly after excision of RAD51 or a RAD51 paralogue, though again not after excision of PIF6 (Fig.1F).*'

2. Why there is no apparent growth inhibition at P2, while DNA synthesis is impaired for RAD51 and RAD51-3 ? This does not make sense to me. Furthermore, the accumulation of DSBs (Fig. 2C) should also lead to growth defects.

Please see comments above and new data on growth in M199 medium: we suspect that HOMEM medium may be more permissive for growth of *L. major* after impairment of important cellular activities.

2. The authors should explain why they have induction of DSBs without RAP especially at 0h (RAD51) and 3-6h ?

The anti-yH2A antiserum always detects some protein, either because of endogenous levels of genome lesions (which may not be limited to DSBs) or because of cross-reaction with unmodified histone H2A. Levels of this background signal vary depending on blot exposure and so we can only ask if there is increased levels of the modified histone after DiCre excision or treatments that result in DNA lesions.

3. Page 6. "KO induction of RAD51 or RAD51-3 resulted in a pronounced increase of γH2A levels in cells navigating through S-phase up to G2/M". The data supporting this claim is missing unless they have FACS analysis of synchronized cultures.

FACS plots of the cells, showing cell cycle progression following release from HU arrest, are shown in Fig.S6.

4. Page 6. "L. major cell cycle checkpoints do not seem to be enacted by the gene KOs". This should be shown experimentally, as this is quite an important point, which would distinguish L. major from higher eukaryotic cells.

We apologise: the wording of this point was too strong. We have altered the text, since we have no way of checking for checkpoints currently.

'Intriguingly, no pronounced changes in cell cycle progression after release from HU arrest were observed upon KO induction of any of the HR genes or PIF6 (Fig.S5), perhaps suggesting cell cycle checkpoints are not enacted by the gene KOs, despite clear DNA damage accumulation after loss of RAD51 or its paralogues.'

5. The genome-wide mutagenesis analysis is very interesting. I think this should be better defined in terms of DNA signatures. For instance, in breast cancer, an analysis of 560 whole genomes showed that 'Signature 3' corresponds to a deficiency in the HR machinery (see Nature 534, 47–54 (2016) and Nat. Med. 23, 517–525 (2017)). Could the authors better define the mutagenesis signature observed after Rad51, Rad51-3, Rad51-4 depletion ? In addition, why RAD51-6 was not included as a control ?

We thank the reviewer for this suggestion. The type of mutation found at each new SNP, with and without HU treatment, is now detailed in new Figs.S8 and S11 (analysis conducted by D.Beraldi; added to author list). To our surprise, we cannot see a difference in the profile of mutation type during growth of the cells with or without DiCre excision, despite changed levels of SNPs and InDels after loss of RAD51 or RAD51-3. It is possible the timeframe of the experiment (6 passages) is insufficient to see a pronounced shift in mutation type due to loss of HR activity. Consistent with this, we cannot map the patterns of mutation we see during *L .major* growth (including of the mutants) to that expected for loss of HR in human cancer (signatures 3 and 8; Nik-Zainal et al, 2016, Nature 534, 47–54). We inserted the following text:

'*Given that excision of RAD51 and RAD51-3 altered the frequency of SNP accumulation, we next examined the types of mutation that resulted in the SNPs (Fig.S8). Though the data showed some bias towards specific forms of base substitution, it was not clear that induced loss of any of the HR genes altered this pattern relative to uninduced controls and nor was any difference seen when comparing loss of RAD51, RAD51-3 or RAD51-4.'*

In addition to looking at mutation signatures, we have re-analysed the SNP and InDel frequency data with greater rigour (Figs.3, 5, S7, S10, S15). To do this, we evaluated the accumulation of mutations during growth in both the DiCre-induced and uninduced cells, and at passage 2 and passage 4, by comparing the genome sequences to the reference genome. Furthermore, we discarded mutations that were common to the two passages in order to exclude variation in the genomes of our cell lines relative to the *L. major* reference genome. We feel this more complete analysis provides the reader with a clearer picture of the consequences of loss of the HR factors. More importantly, this analysis reinforces two findings. 1. Loss of RAD51 or RAD51-3, which affects DNA synthesis, causes a different level of

mutagenesis than loss of RAD51-4, which does not affect DNA synthesis. 2. Loss of RAD51 causes effects on mutation around predicted replication origins that are not seen after loss of RAD51-3, consistent with the different effects of the gene KOs on replication mapping by MFA-seq.

We have incorporated all the above new analyses by substantial rewriting of the section entitled '*Ablation of RAD51 or RAD51-3, but not RAD51-4, results in widespread mutagenesis*'

We did not sequence the genome of cells after excision of *RAD51-6*, I'm afraid.

6. Rad51 is known to physically interact with the replicative MCM helicase in both humans and yeast (Biochem. Biophys. Res. Commun. 2005, 329, 1240–1245; Mol. Cell. Biol. 2008, 28, 1724–1738). It would be important to show that there is a direct interaction with RAD51 and DNA replication components by using immunoprecipitation from their HA-tagged Rad51 strain in hand. This would reveal whether the link between RAD51 and the DNA replication machinery is direct or indirect.

We cannot do this analysis, unfortunately. Although we can add an HA epitope to RAD51, and can thus follow loss of expression after excision, the tag impairs RAD51 function (Fig.S3). Thus, it is likely that interaction analysis would be compromised. Hence, all our experimental analysis was conducted using a floxed and untagged version of the RAD51 gene. Though we have polyclonal antiserum against *T. brucei* RAD51, this does not recognize the *Leishmania* orthologue efficiently (unpublished).

7. In the same line of thoughts it is shown that loss of RAD51 leads to a more pronounced decrease in replication initiation activity at the main replication origins. Can the authors ChIP RAD51 at these origins ?

Please see response above: the HA tagged protein is functionally impaired, so ChIP would be compromised, and we have no antiserum that reacts efficiently with *Leishmania* RAD51.

Minor comments

Fig. 2B. All panels. IdU Fluorescnec should read Fluorescence

Corrected

Reviewer #2

Damasceno et al. used a CRISPR/cas9 and DiCre combined approach to conditionally knockout all RAD51 paralogues (including RAD51, RAD51-3, 4, and 6) in Leishmania and characterized the mutant phenotypes. None of the RAD51 paralogues is essential, although they display cell growth defects after a long-term cell culture. RAD51 paralogue ko cells accumulate DNA damage. RAD51 and RAD51-3 cells

showed decreased level of IdU-incorporation. RAD51, RAD51-3 and RAD51-4 ko cells showed an increased level of SNP. Finally, a different ori firing pattern has been observed in RAD51 ko cells.

The authors have generated a large amount of experiment data, most are of high quality. However, several major conclusions are debatable and can have alternative explanations.

Major issues:

1. None of the RAD51 paralogues showed any growth defects immediately after they are deleted. By definition, these genes are not essential. Although ko cells showed growth defects after 15 passages, this can easily be explained by the fact that these cells are accumulating a large amount of DNA damage since the major DNA damage repair pathway is defective.

We appreciate the reviewer's comments, and have tried to ensure that our discussion accurately reflects this possibility:

'*we show that loss of any RAD51-like gene is not immediately lethal to promastigote L. major but is increasingly detrimental over time, with phenotype penetrance dependent on cultivation conditions.*'.

'*we show that excision of any of these HR factors has no immediate impact of parasite fitness, but rather causes a progressive slowing of growth, presumably due to accrual of problems. Nonetheless, it is clear that Leishmania null mutants of RAD51 (in L. infantum)[30], and the RAD51 paralogues RAD51-4 and RAD51-6 (this work, in L. major), can be generated. Similarly, null mutants of each RAD51-related gene have been described in T. brucei [44, 46]. It is possible such variation in importance reflects species-specific aspects of HR function. However, it is also conceivable that conventional, two step transformation approaches to generate null mutant clones allows for selection of cells possessing compensatory changes that lead to survival - an adaptation that may not always be recovered when using CRISPR-Cas9 to simultaneously ablate both alleles [48], or that emerged in the timeframe we have used during conditional gene excision (this work)*'

In other words, we suggest that while loss of some genes might be immediately lethal, others may only display lethality after secondary consequences of their loss, such as through increased mutation.

In addition, we now provide data on growth of the cells after DiCre-mediated excision in M199 medium, where we document earlier arising defects (Fig.1F). These data reinforce the suggestion that loss of RAD51-like proteins in *L. major* can be detrimental, though the severity is context-dependent.

2. KO of RAD51 changed the ori firing pattern and ko RAD51-3 showed a similar change to a lesser degree. However, this can simply because ori choice is affected by DNA damage (the amount and/or the type of DNA damage). The authors need much better controls if they want to conclude that RAD51 plays a role in DNA replication directly. What about ori firing pattern in a mutant that generates comparable level of DNA damage as RAD51 ko? Will this result in the same change of ori firing pattern?

With respect, this control is provided by KO of *RAD51-3*. In three phenotypes loss of RAD51 and RAD51-3 are highly comparable: reduced growth rate (Fig.1F), increased overall levels of DNA damage (Fig.1E), and impaired incorporation of IdU (Fig.2A,B). Despite this, whereas loss of RAD51 has a clear effect on the DNA replication program mapped by MFA-seq (reduced central peak in each chromosome, increased subtelomere peaks), loss of RAD51-3 causes no such obvious changes, a finding consistent with other evidence for diverged functions of the two HR factors (different pattern of damage in the mutants during the cell cycle, and different levels and patterns of mutation in the mutants during growth).

The IdU incorporation level is lower in RAD51 and RA51-3 ko cells. However, if DNA replication is truly affected, how can cells grow for more than 15 passages without showing any growth defects?

Please see comments above, and new data on growth in M199 medium: HOMEM medium may be permissive for growth of cells lacking some important cellular activities.

In fact, the current observed phenotypes (seemingly DNA replication defects) can have alternative explanations, and the effects of RAD51 and RAD51-3 are likely indirect. For example, accumulated DNA damage may affect ori choice. It is also possible that in RAD51 ko cells, a special type of DNA damage accumulates that preferentially inhibits DNA replication. Additionally, gene involved in DNA replication may accumulate mutations. These possibilities need to be carefully considered.

We agree that the effects may be indirect and have only suggested the (remote) possibility of RAD51 directly acting at origins as one amongst a number of explanations for the data. We trust that rewording throughout the discussion gives sufficient weight to multiple potential explanations for the data.

As regards DNA damage, please see the improved analysis of mutation levels and distribution (Figs.3, 5, S7, S10, S15), as well as new analysis of mutation signatures (Figs.S8, S11). Of note, the latter data does not reveal a specific mutation in the RAD51 KO cells relative to the RAD51 paralogues, but of course this cannot exclude a specific lesion arising in these mutants that is not detected by this approach.

3. Treating cells with 5 mM HU for 8 hrs is quite extreme. HU is known to cause DNA damage directly at high levels, and Figure 2 gH2A western confirmed that cells have experienced much more DNA damage with HU treatment even without RAD51 paralogue deletion. Therefore, the authors are looking at DNA damage responses in addition to DNA replication stress under this condition. When interpreting the data, this should be considered.

We appreciate this point and thank the reviewer. To explore this question, we have examined in greater depth the mutation levels and patterns seen after exposure and release from HU (Fig.S10), as well as the mutation profile (Fig.S11). The conclusion from this work is that, within the timeframe of this analysis, mutation dynamics are not dramatically altered by HU treatment, with one exception: induced loss of RAD51 reduces the level if SNPs after HU relative to loss of RAD51 without HU. This singular change seems consistent with altered DNA replication dynamics in RAD51 mutants and not in RAD51-3. We have described these new data in the results section '*Ablation of RAD51 or RAD51-3, but not RAD51-4, results in widespread mutagenesis*'.

4. Figure 1 showed the cell concentration is lower with prolonged cell culture in RAD51 paralog ko cells. This does not necessarily mean that the cells are having a growth defect. These cells may be more sensitive to quorum sensing factors, for example. The evidence for cell growth defect is not solid in this case.

I'm afraid we are unaware of any published work that suggests *L. major* promastigotes employ quorum sensing, and nor have we seen work in *T. brucei* showing that recombination activity influences the quorum sensing process that dictates growth dynamics in the mammal.

Reviewer #3:

Manuscript shows an analysis of null mutants lacking RAD51 and paralogous concerning DNA replication. The first conclusion that can be taken by results is that null mutantants of the RAD51 paralogues RAD51-4 and RAD51-6 can be generated in L. major. Another conclusion is that conditional gene KO loss of RAD51, RAD51-3 and RAD51-4 led to genome-wide increases in SNPs, demonstrating a widespread role for these related proteins in Leishmania genome stability. I agree with authors about these conclusions. Then, the manuscript discusses the role of RAD51 paralogous onto DNA replication. Although data are very well presented I cannot understand the interpretation of some data proposed by Damasceno et al.

Introduction: Damasceno et al say that "origin number and distribution in Leishmania is unusual, since one study detected only a single site of DNA replication initiation per chromosome [39], while a later study suggested >5000 sites [40]. These data indicate either a pronounced paucity or huge overabundance of origins relative to all other eukaryotes. Alternatively, the disparity in the datasets may be due to a widespread, unconventional route for initiation of DNA synthesis, acting alongside a small number of conventional origins, perhaps indicating novel strategies for DNA replication that may link with genome plasticity."

This proposed alternative is based on the premise that MFA-seq (methodology used in ref 39) would detect conventional origins while SNS-seq (methodology used in ref 40) would detect an unconventional initiation. I can not see the rational to explain these differences.

We apologise for the lack of clarity. We have tried to improve the text:

'*Alternatively, since in neither study was DNA replication mapping correlated with binding of replication initiation factors, the disparity between the datasets may be due to a widespread, unconventional route for initiation of DNA synthesis acting alongside a small number of conventional origins, perhaps indicating novel strategies for DNA replication that may link with genome plasticity*'.

Results:

Figure 3C: you say that "the smaller chromosomes accumulated a higher density of new SNPs than the larger chromosomes". It is difficult to see it in the figure. A graph of correlation containing chromosome size X SNP together with the correlation coefficient (r value) is more indicated to allow this kind of conclusion. Still concerning this figure, authors say that "If not gene expression, then the chromosome size-dependence of SNP density might reflect the limitations of predominant DNA replication initiation at a single origin". If there is a sinlge origin in every chromosome, why short chromosomes would present more SNP?

As all the figures have a lot of panels already, we trust it is acceptable to not add a graph of correlation between chromosome size and mutation, since it is a relatively small observation in the paper. Moreover, we do not have an explanation and have merely noted to trend for completeness.

Figure S10: * is not explained in the legend. Actually, the entire figure is complicated and difficult to understand.

Please see the following text to explain the labels (Fig.S10 is now S13); we used the same text in Fig.S4:

'(*) and (**), GOIFlox and GOIFlox after excision, respectively.'

Figure S13 is lacking.

This figure is now Fig.S16.

Since both RAD51 and RAD51_3 are involved with DNA replication (since both KO affect IdU incorporation), I did not understand why authors did not check the mutant without both proteins. Would be these mutants able to replicate?

We do not know if such a double mutant would be able to replicate, but we provide evidence that the two factors act differently in genome maintenance: different pattern of damage in the mutants during the cell cycle, and different levels of mutation in the mutants during growth.

How authors explain the role of RAD51_3 in DNA replication since lacking of this protein does not change origin profile?

We do not know in detail, but suggest the possibility that RAD51-3 has an important role in tackling stalled replication forks:

'*In the case of RAD51-3, the very modest loss of replication at the main origin, and no clear increase in subtelomeric replication, seems most readily explained by a widespread impairment in genome replication, perhaps because the RAD51 paralogue is needed to guide processes involved in promoting replication in the face of genome-wide impediments. In this regard, the replication phenotypes after loss of RAD51-3 may be comparable with effects seen after mutation of T. brucei MCM-BP [66], a poorly understood factor that modulates activity of the replicative MCM helicase [67, 68]. In addition, such a role for L. major RAD51-3 may be akin to roles for mammalian Rad51 paralogues on stalled DNA replication forks*'.

Discussion:

Lacking of RAD51 causes impaired nucleotide uptake with loss of efficient DNA replication initiation at the single primary origin in each chromosome and increased subtelomeric DNA replication initiation. Therefore authors argue that increased replication from the subtelomeres is insufficient to compensate for loss of the primary initiation events. However, Damasceno et al are not considering that fork speed can be altered in the absence of RAD51. It hs been already been showed that disrupted functions of RAD51 will lead to a reduced replication fork speed (RADIATION DNA DAMAGE AND USE IN CANCER/ THERAPEUTICSTRANSLATION OF RADIATION MODIFIERS E. Dikomey*, K. Borgmann*, S. Köcher*, M. Kriegs*, W. Mansour*, A.C. Parplys*, T. Rieckmann*, **, K. Rothkamm* In: in DNA Repair in Cancer Therapy (Second Edition), 2016)

We thank the reviewer for pointing this out. We have added the following text:

‘A distinct suggestion for an indirect role of RAD51 is that the recombinase does not play an origin- or centromere-focused role, but instead is needed to support DNA replication genome-wide, given previous suggestions that replication from a single major origin in each Leishmania chromosome would be insufficient to replicate all chromosomes in S-phase [39, 41]. Such a function could be compatible with origin-independent roles of HR-directed replication (discussed above) and, in the absence of RAD51, complete chromosome replication is lost during S-phase, leading to impaired mitosis and reduced numbers of cells that license the main origins. Alternatively, loss of RAD51 may slow progress of replication forks emanating from the single central origin in each chromosome, preventing completion of chromosome duplication in S phase. Either scenario may explain the increased levels of MFA-seq reads in the subtelomeres.’

Despite including the above phrase, we ask that the reviewer considers new data (Fig.6, Fig.S16), where we have performed MFA-seq in induced and uninduced RAD51 and RAD51-3 mutant cells exposed to 5 mM HU for 8 hours. These new data suggest that the altered program of DNA replication in the RAD51 mutants, detected by MFA-seq in the absence of HU, cannot be explained simply by replication stress, since the changed MFA-seq profile in the HU-treated cells is dramatically different.

The discussion that RAD51 could be the initiator in the replication process is very, very speculative. I believe that any propose in this direction should arrive after demonstration that (i) ORC KO replicates well and (ii) RAD51 localizes with MFA-seq origins.

We agree this is unlikely, and have stressed this as follows:

‘Such an origin-specific function for RAD51 or RAD51-directed HR has no precedent and, indeed, would be distinct from origin activity in T. brucei, where these sites are conventionally defined by binding ORC’.

In addition, we have explicitly pointed out that the experiments are needed to test such a possibility:

‘Therefore, it remains to be tested if RAD51 associates with DNA replication initiation complexes and/or with the origins of replication.’

Moreover, we have provided, after these statements, several potential explanations that we consider more likely. Nonetheless, we wish to stress that this is the only study we are aware of where DNA replication dynamics have been shown to be altered by loss of an HR factor, so we feel it is legitimate to explore the various potential explanations for the finding.

As we discuss above, we cannot currently map RAD51 binding in the *L. major* genome. In addition, we are currently trying to map ORC binding, as well as evaluate the consequences of ORC loss, but have no data yet.

The increment of origins after RAD51 KO could be due the fact that: single main origin conflicts with transcription (or some genomic structure), RAD51 is absent to fix and solve stalled/collapsed forks; then dorment origins are fired. I did not understand why this simplest interpretation was not considered.

We did not consider activation of dormant origins because we cannot detect increased MFA-seq signal in the mutants that localizes to SSRs, where we might expect such origins to be found, based on the single MFA-seq peak in each *L. major* chromosome and on precedent in *T. brucei* (Tiengwe et al, 20102, Cell Reports). However, it is possible that subtelomere MFA-seq signal in the mutants represents activation of dormant origins, though we have no evidence these regions bind ORC. We have therefore added the following statement:

'*Alternatively, subtelomeric DNA synthesis may result from the activation of dormant origins in these regions, serving as back-up for the primary SSR-focused DNA replication initiation reaction; thus, when the primary reaction is compromised by loss of RAD51, subtelomere initiation assumes greater importance*.'

Minor issues:

1. Adding line numbers will be more convenient for the reader.

Our apologies for this oversight: line numbers have been added.

2. Page 7, first line, "promoting effective DNA synthesis" is not appropriate based on current data.

I'm afraid we disagree, since the data measures incorporation of IdU and therefore evaluates levels of DNA synthesis (either replicative or repair)

3. Page 7, second line "during S phase" – what evidence is there to support that DNA damage only occur in S phase in the ko cells?

These data are shown in Fig.2C, where we show levels of yH2A increase as cells progress through S-phase (as evaluated by FACS; Fig.S6).

4. Page 8, in the section "generation of conditional double gene…", first line "implicating only RAD51 and … in DNA synthesis" is not an accurate statement based on current results. The defects in DNA synthesis can well be indirect, from accumulated DNA damage.

We agree that the effects may be indirect, but the statement is consistent with only loss of these two factors reducing incorporation of IdU (a measure of DNA synthesis).

5. Page 9, second paragraph from top, last third line, “suggestion that RAD51-3 alone … DNA replication” again is not an appropriate statement based on the current data. See reason listed above.

We have altered the text:

‘*However, a signal reduction was detected 72 h after RAD51-3 KO induction in both the RAD51-4 and RAD51-6 null backgrounds, consistent with loss of only RAD51-3 amongst the paralogues impeding DNA synthesis*.’

6. Page 9, in the paragraph above the section heading “Loss of RAD51…”, the 7th line from top, “that” should be “than”.

Corrected

7. Page 10, last paragraph, line 5 from top, “Fig. S12” should be “Fig. S13”.

All figure citations have been changed.

8. Discussion needs to be revised considerably not to extrapolate and propose too much based on the available data.

Please see changes listed described in the discussion.
