# Supplementary material for: Conditional knockout of RAD51-related genes in Leishmania major reveals a critical role for homologous recombination during genome replication
Source: PLoS Genet. 2020 Jul 1;16(7):e1008828. doi: 10.1371/journal.pgen.1008828 (PMC7360064; doi:10.1371/journal.pgen.1008828)
Supplement: Attachment — Submitted filename: Critiques.docx [file pgen.1008828.s018.docx]

RE: PGENETICS-D-19-01903

Title: Conditional knockout of RAD51-related genes in Leishmania major reveals a critical role for homologous recombination during genome replication

Authors: Damasceno et al.

Comments:

Damasceno et al. used a CRISPR/cas9 and DiCre combined approach to conditionally knockout all RAD51 paralogues (including RAD51, RAD51-3, 4, and 6) in Leishmania and characterized the mutant phenotypes. None of the RAD51 paralogues is essential, although they display cell growth defects after a long-term cell culture. RAD51 paralogue ko cells accumulate DNA damage. RAD51 and RAD51-3 cells showed decreased level of IdU-incorporation. RAD51, RAD51-3 and RAD51-4 ko cells showed an increased level of SNP. Finally, a different ori firing pattern has been observed in RAD51 ko cells.

The authors have generated a large amount of experiment data, most are of high quality. However, several major conclusions are debatable and can have alternative explanations.

Major issues:

1. None of the RAD51 paralogues showed any growth defects immediately after they are deleted. By definition, these genes are not essential. Although ko cells showed growth defects after 15 passages, this can easily be explained by the fact that these cells are accumulating a large amount of DNA damage since the major DNA damage repair pathway is defective.

2. KO of RAD51 changed the ori firing pattern and ko RAD51-3 showed a similar change to a lesser degree. However, this can simply because ori choice is affected by DNA damage (the amount and/or the type of DNA damage). The authors need much better controls if they want to conclude that RAD51 plays a role in DNA replication directly. What about ori firing pattern in a mutant that generates comparable level of DNA damage as RAD51 ko? Will this result in the same change of ori firing pattern?

The IdU incorporation level is lower in RAD51 and RA51-3 ko cells. However, if DNA replication is truly affected, how can cells grow for more than 15 passages without showing any growth defects?

In fact, the current observed phenotypes (seemingly DNA replication defects) can have alternative explanations, and the effects of RAD51 and RAD51-3 are likely indirect. For example, accumulated DNA damage may affect ori choice. It is also possible that in RAD51 ko cells, a special type of DNA damage accumulates that preferentially inhibits DNA replication. Additionally, gene involved in DNA replication may accumulate mutations. These possibilities need to be carefully considered.

3. Treating cells with 5 mM HU for 8 hrs is quite extreme. HU is known to cause DNA damage directly at high levels, and Figure 2 gH2A western confirmed that cells have experienced much more DNA damage with HU treatment even without RAD51 paralogue deletion. Therefore, the authors are looking at DNA damage responses in addition to DNA replication stress under this condition. When interpreting the data, this should be considered.

4. Figure 1 showed the cell concentration is lower with prolonged cell culture in RAD51 paralog ko cells. This does not necessarily mean that the cells are having a growth defect. These cells may be more sensitive to quorum sensing factors, for example. The evidence for cell growth defect is not solid in this case.

Minor issues:

1. Adding line numbers will be more convenient for the reader.

2. Page 7, first line, “promoting effective DNA synthesis” is not appropriate based on current data.

3. Page 7, second line “during S phase” – what evidence is there to support that DNA damage only occur in S phase in the ko cells?

4. Page 8, in the section “generation of conditional double gene…”, first line “implicating only RAD51 and … in DNA synthesis” is not an accurate statement based on current results. The defects in DNA synthesis can well be indirect, from accumulated DNA damage.

5. Page 9, second paragraph from top, last third line, “suggestion that RAD51-3 alone … DNA replication” again is not an appropriate statement based on the current data. See reason listed above.

6. Page 9, in the paragraph above the section heading “Loss of RAD51…”, the 7^th^ line from top, “that” should be “than”.

7. Page 10, last paragraph, line 5 from top, “Fig. S12” should be “Fig. S13”.

8. Discussion needs to be revised considerably not to extrapolate and propose too much based on the available data.
